# Experiences and views of older people on their participation in a nurse-led health promotion intervention: “Community Health Consultation Offices for Seniors”

**DOI:** 10.1371/journal.pone.0216494

**Published:** 2019-05-13

**Authors:** Anne Esther Marcus-Varwijk, Dónya S. Madjdian, Emely de Vet, Monique W. M. Mensen, Tommy L. S. Visscher, Adelita V. Ranchor, Joris P. J. Slaets, Carolien H. M. Smits

**Affiliations:** 1 Research Group Innovating with Older Adults, Windesheim University of Applied Sciences, Zwolle, The Netherlands; 2 Department of Internal Medicine, University Medical Center Groningen, University of Groningen, Groningen, The Netherlands; 3 Chair group Strategic Communication, Sub-department of Communication, Philosophy and Technology: Centre for Integrative Development, Wageningen University, Wageningen University and Research, Wageningen, The Netherlands; 4 Research Group for Healthy Cities, Windesheim University of Applied Sciences, Zwolle, The Netherlands; 5 Health Psychology Section, University Medical Center Groningen, University of Groningen, Groningen, The Netherlands; 6 Leyden Academy on Vitality and Ageing, Leiden, The Netherlands; Nord University, NORWAY

## Abstract

**Background:**

The growing number of community-dwelling older adults and the increased risks of adverse health events that accompany ageing, call for health promotion interventions. Nurses often lead these interventions. The views and experiences of older adults participating in these interventions have rarely been studied. To understand the views of targeted older adults, qualitative studies are essential. The aim of this study was to investigate the views and experiences of older adults on their participation in a nurse-led intervention, taking into account their views on healthy aging.

**Methods:**

In a qualitative study, nineteen Dutch older adults aged 62 to 92 years participated in semi-structured interviews. These were transcribed verbatim and coded with the Qualitative Data Analysis Miner software program. The Qualitative Analysis Guide of Leuven was used for data analysis.

**Results:**

Based on the analysis of the interviews, the following main themes emerged from the data reflecting the experiences of the participants: 1) awareness of aging, 2) experienced interaction with the nurse, and 3) perception of the consultations as a check-up and/or personal support.

**Conclusions:**

This study underscores the importance of nurse-led interventions that match older adults’ personal views concerning healthy living, and their views and experiences concerning these interventions. Older adults’ holistic views of healthy living were not always assessed and valued by the nurses. Also, our study shows a wide variety of expectations, views and experiences among the participating older adults. This implies that health professionals should adjust their working and communication methods to the older adult’s views on life.

## Introduction

Health promotion and disease prevention among the growing group of older adults is a significant challenge as ageing is often accompanied by increased vulnerability [1–3]. Consequently, early interventions to promote healthy behavior and, to preserve or improve daily functioning in community-dwelling older adults are needed [4,5]. Health promotion and disease prevention have formed part of nursing practice tasks since the 20th century [6–9]. Currently, nurses in primary and community care are increasingly involved in health promotion and disease prevention programs aimed at maintaining or improving daily functioning in community-dwelling older adults [10]. To carry out these programs, nurses should be equipped with and utilize the knowledge and skills related to: a) lifestyle and behavior change, b) assessment, c) communication, and d) advocacy [6].

Taking all these skills into account, health promotion interventions should be tailored to the individual older adult’s needs and concerns and ensure their voices are heard [11]. In theory, person-centered communication approaches such as shared decision making (SDM) and motivational interviewing (MI) can facilitate nurses in delivering tailored interventions [12]. Taking the older adults’ perspective into account is a vital element when executing tailored interventions [12, 13]. SDM and MI provide opportunities to enable this by encompassing elements such as respecting autonomy, developing trust, maintaining equal power balance, showing understanding and empathy, listening, and providing professional advice [12, 14, 15]. In this way, SDM and MI enable nurses to encourage older adults to explore their values and preferences in order to provide behavioral change counseling [12].

Despite significant efforts to teach person-centered communication such as SDM in nursing training implementation in practice has proven difficult [16, 17]. For example, in practice, nurses might not always be able to identify the older adults’ individual needs because nurses experience the older adults’ needs as vaguely expressed by their clients, which could lead to misinterpretations or missed opportunities to tackle challenges [18].

Moreover, most studies have focused particularly on the interaction between the physician and the client in the context of clinical practice rather than on the interaction between the nurse and the older adult in the context of health promotion, taking into account the older adults’ personal environment [15, 19–21]. Furthermore, studies that have focused on nurse–client interactions often do so from a nurse’s perspective rather than from the older adult’s perspective. Only a few studies address the nurse–older adult interaction from the client’s perspective, although these often take place in a clinical setting [19]. For instance, a study that addressed barriers in nurse–client interactions from the older adults’ perspective in a hospital setting showed that common barriers were mostly related to communication (using medical terms and sudden changes of subject), attitudes (insincere or authoritative), and unfriendliness [22]. Moreover, studies evaluating health promotion and disease prevention interventions mainly use quantitative measurements or designs and do not clarify the experiences and views of the older adults themselves [23].

To create a broader perceptiveness within nurse-led health promotion and preventive interventions, and to understand the views of targeted older adults, qualitative studies are essential [24]. The aim of this study was to investigate the views and experiences of older adults on their participation in a nurse-led health promotion intervention in the Netherlands, taking into account their views on healthy aging.

## Methods

The *“Community Health Consultation Offices for Seniors (CHCO)”*, a Dutch nurse-led health promotion intervention, was used as a research setting. The current study adopted a qualitative design involving semi-structured interviews with community-dwelling older participants to answer the following research question: *‘What are the views and experiences of older adults on their participation in the nurse-led CHCO intervention*, *taking into account their views on healthy aging*?*’* Ethical approval for this qualitative study was granted by the ethics review board of the University Medical Center Groningen (M13.137223) and written informed consent was obtained from all participants.

### The CHCO intervention

The CHCO intervention for seniors is a nurse-led intervention targeting community-dwelling older adults aged 60 or older, with augmented risks of adverse health outcomes [25]. Older adults received an informational letter about the intervention if they were members of the participating care association and insured by the largest health insurance company in the region. A selection procedure was implemented to select vulnerable older adults. Subjects were invited to participate in the CHCO-intervention, when meeting at least one of the following criteria: 1) a frailty score of >3 measured by the Groningen Frailty Indicator, 2) being overweight (<70 years BMI > 25 kg/m^2^ and/or >70 years BMI > 30 kg/m^2^) and, 3) currently smoking. The Groningen Frailty Indicator (GFI) is a widely-used multidimensional screening instrument to identify frail older adults [26]. It consists of fifteen self-reported items with an overall sum-score of 15. A person with a total of 3 points or more is considered frail [27, 28].

Participants who agreed to participate in the intervention were asked to fill in a comprehensive health assessment questionnaire before meeting with the community health nurse. During the first consultation with the nurse (60 minutes), the nurse checked the assessment questionnaire and, if agreed, biometric measures were performed. Based on this information, the nurse was able to provide tailored advice and refer people to other health professionals (such as the general practitioner), if needed to support daily functioning or enhance health outcomes. After the first consultation, a follow-up (20–30 minutes) and annual consultation (60 minutes) would follow if necessary. Further details about the CHCO intervention are described in detail elsewhere [25].

### Sampling

A total of fifteen interviews with individuals and two with married couples were conducted from January 2014 to March 2014. A purposive sampling strategy was used to recruit participants for the interview [29]. Thirteen Consultation Offices were selected based on: diversity in their geographical locations (urban versus rural); establishment of consultation office (new location versus established); and diversity in characteristics of the nurses running the consultation offices. The aim was to interview at least one older adult per Consultation Office for which we randomly sampled a maximum of 6 older adults per office. Selection was based on whether they had participated in the intervention at least once and to reduce recall bias, if they had received the (most recent) consultation between November 2013 and February 2014, which means that there would be a maximum of 4 months between their participation in the intervention and the interview.

We invited a total of 61 older adults to take part in this study by mail. After receiving the letter, the interviewer (DM) phoned these older adults to explain the study, invite them to participate and, if interested, to double-check whether they met inclusion criteria. We were not able to get in touch with 21 older adults. Out of the 31 who answered the phone, twelve declined to participate. Their reasons included: not interested, no time, never went to the consultation, or could not recall the consultation well enough for an interview. Nineteen participants agreed to participate in this study, including those participating in a pilot interview, covering all thirteen consultation offices. In three locations, more interviews were planned. No new concepts arose from the data after 19 interviews, therefore, we decided not to contact the other nine people on the list, as data saturation had been reached.

### Interview procedure

A pilot interview was conducted by the interviewer (DM) and an observer (MM). The research team discussed this interview (CS, AM, MM, DM) to verify 1) whether the topic guide met the requirements for this particular study, 2) if the questions and formulation of concepts were suitable for the older adults to understand the actual content of the research, and 3) to evaluate interview techniques. Only small changes were made to the topic guide after the evaluation of the pilot interview. The research team members agreed that the pilot interview included valuable information, and since only minor adjustments were made to the topic guide (i.e. more probing questions, changes to the order of questions in order to create a better flow) the pilot interview was added to the data analysis.

Before starting the interview, participants were informed by means of a written consent. At the start of the interview, the interviewer asked a number of introductory questions intended to improve recollection of the nurse consultations. Subsequently, specific topics were discussed during the interview, see Table 1 for the interview guide.

**Table 1:** Interview guideline about the experiences and views of participants on the CHCO intervention.

Theme	Questions
Introduction	What can you recall from the consultations?
	What was your first thought when you received the invitation to the office?
	Did the consultations meet the expectations you had beforehand (if any)?
Healthy aging	What does healthy aging (or healthy living) mean to you?
Verbal interaction with the nurse(s)	What did you think of the conversations you had with the nurse?
	How would you describe the ambiance during the conversation?
	What did you think of the nurse?
Tailored advice and decision-making	Before the first consultation, how did you try to lead a healthy lifestyle?
	How motivated were you to change certain behaviors (if any)?
	Process: can you tell me something about your first consultation when you talked about your current health status?
	Can you tell me what kind of advice you were given during the consultations?
	Was this advice relevant/applicable to your personal situation?
	How satisfied are you with this advice?
	What did you do with this advice in the end?
	Did you receive an ‘advice sheet’?
	Did you feel you were treated as an equal during the conversations?
	To what extent did you actively play a part in deciding the topic of the conversation?
	Did you have the feeling you could ask or say anything?
	To what extent did you feel the nurse was trying to steer you in a particular direction?
	What qualities do you think a nurse should have?
Effect/impact of consultations	All in all, can you tell me what the conversations meant to you personally?
	Do you now have the feeling you can do more to age healthily than you did before?
	What did you think about the information you received during the conversation?
Closure	If you were invited one more time, would you go again?
	In your opinion, what could be changed?
	Do you have the feeling you could tell me anything you wanted to?

All interviews were conducted in the participants’ homes. The interviews ranged from 30 to 90 minutes (average: 60 minutes). After each interview, a short reflection report about characteristics of the interviewee and the context of the interview were written.

### Data analysis

Interviews were audio-taped and transcribed verbatim, and the Qualitative Analysis Guide of Leuven was used to guide data analysis [30]. The Qualitative Analysis Guide of Leuven (QUAGOL) is a theory and practice-based, systematic, yet flexible guide for analyzing qualitative data. Its main characteristics are constant data comparison and team dialogue during the entire process of data analysis [30]. The analysis consisted of two parts: thorough preparation of the coding on paper, and the actual coding and analysis facilitated by the Qualitative Data Analysis (QDA) Miner v4.1.19 software program.

During the first phase of the analysis after reading and re-reading the interview, short narrative reports were written to capture the essence of the participants’ story of the interview (DM and AM). Additionally, conceptual schemes were developed to answer the research question. To ensure rigor, the research team (CS, AM, MM, and DM) discussed the narrative reports and conceptual schemes of the first six interviews, to develop a comprehensive conceptual scheme, which consisted of the most important data linked to the research aim. Subsequent interviews were compared with previous conceptual schemes, and newly emerging concepts were added. The research team verified whether this extended conceptual scheme was appropriate for answering the research question. During the second phase, DM, MM, CS, and EV discussed the list of concepts, following which the QDA miner software program (the actual coding process) was used to attach codes to the relevant interview fragments. All codes were listed, with an explanation of their meaning and typified quote.

The research team checked abstract and ambiguous fragments to understand whether new codes should be added or whether existing codes should be merged. All discussions resulted in agreement. The codes were grouped into more abstract concepts and, if required, concepts were split into sub-concepts. Two researchers re-read the interviews to verify the accuracy of the storylines and to determine their comprehensibility (DM and AM). The results were structured in a conceptual frame, and themes were developed and discussed within the research team (DM, MM, CS, AM, TV, and JS) to answer the research question. The final framework was agreed upon by all authors.

## Results

Nine men and ten women were interviewed. The average age was 70 years (range = 62–92). Demographic and health status data are presented in Table 2.

**Table 2:** Characteristics of the nineteen participants.

Sex		Age (years)		Marital Status		Health Status	
males	9	60–69	6	Married	9	Current Smokers	6
females	10	70–79	10	Widowed	8	BMI* ≥ 28	10
		80–89	2	Divorced	1	Frailty GFI** ≥ 4	12
		90+	1	In a relationship	1		

*Body mass index (BMI): overweight BMI ≥ 28

**Groningen Frailty Indicator (GFI): identified as frail GFI ≥ 4

From the analysis of the interviews, the following main themes emerged from the data reflecting the experiences of the older participants: 1) awareness of aging, 2) experienced interaction with the nurse, and 3) perception of the consultations as check-up and/or personal support. Table 3 lists the themes, categories and codes that emerged from the analyses.

**Table 3:** Themes, categories and codes of the experiences and views of participants on the CHCO intervention.

Themes and categories	Codes
**Theme 1: Awareness of aging**	
*A healthy lifestyle*	Food and drink
	Mental wellbeing
	Mindfulness
	Alcohol in moderation/no alcohol
	Conscious of unhealthy lifestyle
	Physical activity
	Rest, cleanliness, and regularity
	Sleeping
	Not smoking
	Social contacts
	Mobility
	Being an example to others
	Enjoyment
	Hobby’s
*Dealing with (new) opportunities and constraints in life*	Activate yourself
	Perseverance
	Aspiring to a long life
	Being positive/optimistic
	Anticipation, Acceptance
	Knowledge/life experience
	Nothing is a must, anything goes
	Unpredictability of life
	Seize the day
	Take your responsibility
	Continuing doing the things you always did
	Religious coping activities (e.g. prayer)
	Being a role model for others
**Theme 2: Experienced interaction with the nurse**	
*Evaluation of the nurse’s general skills*	Asking questions versus not asking questions
	Advocacy
	Empathy
	Supportiveness
	Listening, to be taken seriously versus not feeling taken seriously
	Personal approach versus impersonal approach
	Creating a positive atmosphere (friendly, open, making people feel at ease)
	Honesty
	Reflection/evaluation
	Competent nurse versus incompetent nurse
	Connection versus no connection
*Receiving advice*	Receiving information during the consultation
	Content of advice
	Referrals
	Written advice received from the nurse
	Way of advising
	Follow-up
*Decision-making processes*	Participation and power balance
	Respecting autonomy
	Presentation of options and priorities by the nurse
	Exploring personal preferences and options
	Discussing the problem
	Precontemplation (not open to changing behavior)
	Former failures to change behavior
	Keeping true feelings/thoughts to oneself
	Making choices together
**Theme 3: Perception of the consultations as check-up and/or personal support**	
*Perceiving the consultation as a physical check-up*	Focus on physical health
	Control function
	Feeling of security
	Second opinion
	No influence on personal lifestyle
	Positive impact on lifestyle
	Raising awareness
	Confirmation of healthy behavior/health status
	Informative
*Receiving personal support from the nurse*	Focus on mental health
	Good feeling
	Breaking off fixed patterns
	Empowerment
	Feeling of support
	Someone who is listening/being able to tell your story
	Moment of personal contact
	Having someone to fall back on

### Theme 1: Awareness of aging

The main categories to emerge from the data related to the first theme were: 1) a healthy lifestyle and 2) dealing with (new) opportunities and constraints in life.

#### A healthy lifestyle

Most participants stated that their main goal was to enjoy life and to live as long as possible. The participants mentioned a variety of ways to achieve that goal: staying mentally sharp and physically fit, staying mobile and flexible, being a good parent and grandparent, and providing support for family and friends. Some participants mentioned spiritual aspects (prayers, for example) or mindfulness as part of healthy living. One of the participants explained staying mobile and active in daily life as follows:

*“Well, you know, I promised myself that I would go outside every day. I tell myself, don’t sit in your chair all day, don’t even think about it! Don’t just sit there twiddling your thumbs. That’s no way to get a thumbs up (laughs). But anyway*, *chop chop, off we go! Outdoors! Even if it’s only for a carton of milk. Just a quick trip to the supermarket.” (Male participant)*

Participants often mentioned that, from their point of view, healthy aging practices were part of a certain routine, and participants had practiced them for many years. As one interviewee said, for example:

*“Eat healthily, no, just healthy food. Just like, every day. Then sometimes they ask you to fill in what you’ve been eating. Then I think it probably sounds very boring. It’s the same thing every day; it’s just the vegetables that change every day, you know. It’s once a week, it’s macaroni, and I always say Friday is leftover day*, *that’s when we empty out the fridge.” (Female participant)*

#### Dealing with (new) opportunities and constraints in life

Participants mentioned that aging comes with discomforts and constraints (physical, mental, and social), and this reminded the participants that they are growing older. Acceptance and anticipation of these changing situations seemed to be important but having a sense of control over these situations was important too. For example, participants felt a need to rearrange their lives by choosing to take things more slowly or to be more careful. The following quote illustrates this:

*“Puttering around in the garden is my favorite hobby. I will never give that up. Even though I’m in pain, you know? I just dig for a while, and then I sit down for a while*. *Hop, and again. Just carry on. So, in that way you are optimistic, but stubborn too.” (Male participant, who recently had a cardiac bypass)*

### Theme 2: Experienced interaction with the nurse

In this theme, a range of responses about the interaction between the nurse and participants was extracted from the data. The theme consists of three categories: 1) evaluation of the nurse’s general skills, 2) receiving advice, and 3) decision-making processes.

#### Evaluation of the nurse’s general skills

Codes concerning the evaluation of the nurse’s general skills included a broad range of concepts by the participants (see Table 3 for more details). Many participants felt that the nurse created a positive atmosphere during the consultations. They saw the nurse as friendly and professional. Some participants felt obliged to be open and honest about their health concerns with the nurse professional, as that would enable the nurse to assist them better. Participants who experienced the nurses as understanding, involved, and being interested often felt that they were taken seriously and felt comfortable to speak up and share more intimate stories. As one female participant said:

*“That you feel at ease and maybe also eh …talk about things you wouldn't normally talk about, so to speak*. *Not to say we did that, but you felt you could. If there was something that I don’t think I would tell anyone else.” (Female participant)*

Some participants mentioned that they felt a connection, or a match, with the nurse during the consultation. This ‘connection’ was often difficult to put into words and points to an ‘affair of the heart.’ Participants would relate it to perceived equality, a personable approach, or having the same age or hometown. In cases where participants did feel a connection with the nurse, participants mentioned that the nurse had been able to pinpoint underlying problems. On the other hand, some participants also experienced the nurse as impersonal and therefore did not feel a connection with the nurse, independent of the nurse’s perceived professionalism.

#### Receiving advice

The most common advice participants received during the consultations was related to weight loss, physical exercise, and nutrition. Also, participants were given strategies for fall prevention and advice to quit smoking and invest in social contacts. General information on fire prevention, medication, and post- surgery was received as advice incidentally. Some participants could not recall having received any advice. In addition, some participants were referred to the general practitioner, mental health care, or a dietician.

Participants’ experience of how the nurse advised them varies. One advice method, as experienced by the participants, was that the nurse asked several questions about a certain topic. This method was used specifically in case of psychological or emotional issues. By asking questions rather than making proposals, participants felt they had the final say. Since the participants were able to elaborate on their own situation and way of living, the advice received was more tailored to the participant. This kind of advice was taken seriously in most cases. Two examples illustrate this way of advising. The first example is a participant who was unaware that she was retaining fluids due to salt intake. This resulted in increased weight and higher blood pressure, for which she already took medication. After exploring her dietary pattern with the nurse, she was advised to lower her salt intake. After following this advice, the participant reported that her weight and her blood pressure decreased almost instantly. In the second example, the participant discussed her stool problems with the nurse, a (perceived) sensitive issue. She had never told a doctor about these problems. However, she felt that she could trust the nurse, and the nurse asked her the relevant questions and was able to analyze the underlying problem. The following quote illustrates this example:

*“I would never tell any doctor or medical person that I use herbal remedies* .*I would never tell them because I also know it’s bad*. *I just know*. *But yes*, *you also have to go to the bathroom and if you can’t without those things then*, *then*, *you also feel eh*, *rotten*. *Well and I did talk about it then* (to the nurse during the consultation) *and somehow that does give you some confidence or something*, *to talk about it to the doctor*. *If I hadn’t told the nurse*, *then it would still be a problem now*. *Because I wanted to stop but didn’t know how*. *I thought I would never be able to stop* (using these herbal remedies). *” (Female participant)*

Another perceived advice method was related to the nurse proposing the wisest option or the healthiest choice regarding lifestyle issues. Participants said the nurse emphasized that ultimately the participant should decide what to do with this advice. Experiencing this way of advising did not feel compulsory because they felt that their autonomy was respected. Some participants also felt that this advice did not connect to their own preferences. The following quotes illustrate this type of advice (both participants were smokers):

*“Let me put it like this, the advice I got was strongly recommended because the nurse said*, *well, that’s the best way to do it. Well, the nurse gave the impression; if you do it like that it’s wise, and if you don’t it’s foolish. Anyway, the nurse said to me, the choice is yours. The nurse can’t force you.” (Male participant)*

*“She’s doing her job*. *And she also wants to set the right example*, *that it’s better for you not to do it* (smoking). *I get it*. *But it didn’t interest me at all* (laughs).” *(Male participant)*

Finally, there was one case in which the participant experienced the way of advising as patronizing and interfering with her life. The following quote illustrates this:

*“Because the nurse starts nagging again saying I might be a bit on the heavy side or uh, you have to do this or that. That’s what they say. You have to do this and you have to do that, you know, then they are really lecturing you a bit*, *you know.” (Female participant)*

#### Decision making processes

In this category, different aspects of decision making were explored among participants. Experiences concerning decision-making processes differed per case. During the consultations, an extensive questionnaire was used to investigate the participant’s overall health. In many cases, the nurse used the questionnaire to facilitate the consultation. Some participants mentioned that there was ample space for them to ask questions or bring up personal issues. Others felt they had no influence on the content of the consultation. In two cases, participants kept their thoughts/problems to themselves. Reasons for holding back information were related to feelings of shame or the feeling that the issue was not important enough to talk about.

During the consultations, the nurse often pointed out certain health-related risks such as smoking or being overweight. A variety of patterns was visible in the decision making-processes and the resulting potential of behavior change. The first pattern shows participants who deliberately choose to maintain their unhealthy behavior: they know that their behavior is not healthy, but they are not willing to give it up. This pattern was seen in male participants who smoked, some of them having a lung disease. Participants mentioned that the nurse tried to discuss their smoking behavior by saying that smoking is unhealthy. Although they agreed it was unhealthy, they had no intention to quit smoking. They explained that smoking was not a problem to them, that giving up would not benefit their health at their age, or it was all they had left after difficult times. “*Save us both the trouble*, *I won’t quit anyway”*–these participants made clear to the nurse that they would not give up anyway. Nurses took notion of this and often changed the subject. In this way, the participants felt that their autonomy was respected.

The second pattern refers to the participants who show ambivalence towards their health-related behavior. On the one hand, they acknowledge that a specific practice is unhealthy and to some extent they would like to change their behavior. They are aware that changing their behavior will be beneficial to them. On the other hand, these participants had tried to change that behavior before without a positive result. In addition, these participants did not experience significant problems in their daily functioning despite related health problems such as high blood pressure or high cholesterol. This pattern mostly emerged when talking about weight loss. During the consultations, they were mostly given general advice by the nurse and no tailored action plan was formulated or specific decision made.

In a third pattern, two participants said they recalled that the consultations with the nurse motivated them to change their behavior. They shared decision-making and talked about a specific action plan to change. Both cases were about weight loss. In one case, the nurse started by finding out more about the cause of the problem, proposed several options, listened to the participant’s considerations and asked the participant to consider other options. This participant was made aware, found a solution by herself and had lost some weight. The other participant described how he and the nurse discussed being overweight, both contributing equally to the conversation. While the nurse started the discussion by letting the participant ‘face the facts’ in a friendly manner, and created awareness about the risks of being overweight, the participant had the opportunity to talk about his previous experiences on losing weight and his personal considerations. In anticipation of this information, the nurse proposed some options and recognized the participant’s personal considerations concerning these alternatives. At the end of the consultation, one suitable option was left and the participant decided to try this option. A realistic goal was set by agreement, which was to be achieved by the next consultation. The following quote illustrates the result of a participant’s motivation and the goal he had set together with the nurse:

*“Because you don’t want to look like a fool*. *If you make a promise and you don’t follow through*, *that just doesn’t feel right*, *does it*? *And I mean*, *if you go somewhere*, *then you have to make a promise*. *Then you have the motivation to say*, *‘well*, *I did promise the nurse*. *Well*, *then I just have to*.*’ I’m thinking*, *‘well*, *I can manage this*, *you know*.*’ I’m thinking*, *‘I’m going to do this*,*’ you know*. *Because that was also my goal*. *I’m thinking*, *‘well that eh*, *we can make this happen*.*’” (Male participant)*

### Theme 3: Perception of the consultations as check-up and/or personal support

In this final theme, participants describe how the intervention did or did not add value to their lives. The theme consists of the following categories: 1) perceiving the consultation as a physical check-up, and 2) receiving personal support from the nurse.

#### Perceiving the consultation as a physical check-up

Participants experienced the consultations as a physical check-up, advice, or a second opinion on their health status. This is illustrated by the following quote:

*“Yes, sufficient. That check-up is more than sufficient*.*” (Male participant)*

The feeling that someone is keeping an eye on them as they age was strong, even when they were already under the supervision of other health care professionals such as general practitioners, practice nurses, medical specialists, and physiotherapists. Some participants reported small positive changes concerning their physical health because they had received tailored advice on that subject. There were also participants who mentioned that they appreciated the physical check-up but noted that they were not interested in lifestyle advice.

#### Receiving personal support from the nurse

In contrast to those who experienced the CHCO intervention as just a physical check-up, other participants mentioned that the personal added value related more to their psychosocial wellbeing. Participants said having the possibility to talk to someone who took the time for them meant much more than the physical check-up. Participants explained that usually a “consult” with their general practitioner (GP) was approximately 10 minutes. They explained that they did not want to bother the GP with their problems because, in their opinion, the GP’s time seemed so valuable. The average duration of the nurse-consultations was approximately 60 minutes, with five cases lasting less than 30 minutes. Participants mentioned that they appreciated the time the nurses took to listen to them. Interestingly, older adults over 80 years old indicated that the moment of personal contact and the opportunity to talk to someone felt nice and pleasant. They did not experience the consultations to be beneficial to their psychical health but enjoyed the personal contact, as illustrated by the quote of an 85-year-old participant:

*“Added value..Not really … Well, I think if it’s a nice chat*, *then that’s okay right?” (Female participant)*

In addition, some participants mentioned that they felt a connection with the nurse. They felt that the nurse struck the right chord and helped them to see underlying problems. For example, one participant talked about her husband who suffered from depression. During the consultation with the nurse, she could talk about the influence her husband’s depression had on her daily life. After the consultation, she felt supported and strengthened to deal with this issue. The following quote illustrates this example:

*“Yes, we did talk about my husband. That’s real attention, you know. Because, I was suffering because of that. But I never noticed that I was suffering actually. That it was such a big part of my life you know? But you know, you do have to from outside, they have to find your right, well, the right triggers, you see? It almost happens by chance*, *because not everyone is the same as I am. Despite me being pretty chatty myself, but at a certain point when you’ve been living with a depressed husband, you get pulled into that process gradually, you know. And that has just been a breakthrough, that I didn’t have to do that. That I should find another way to deal with that.” (Female participant)*

## Discussion

This study contributes to the understanding of the “black box” of health promotion and disease prevention by providing in-depth information and rich data about the views and experiences of older adults in the context of health promotion in a primary care setting. The diverse representation of older adults expressing a variety of expectations and views reflects a heterogeneous group. The results showed a large variety in experiences and views regarding the following themes that emerged from the data: 1) awareness of aging, 2) experienced interaction with the nurse, and 3) perception of the consultations as check-up and/or personal support.

Older adults appeared to have a holistic view on health. Healthy living included being physically, mentally, and socially active. In addition, they addressed that as they grew older, they felt the need to anticipate new opportunities and constraints in life that were unique to every single person. Having a sense of control over these situations was considered important. These findings relate to other studies that show that (successful) aging encompasses acceptance and self-acceptance, as well as engaging in life; for example, in maintaining social relationships [31–33]. Moreover, the relatively new formulation of health as “*the ability to adapt and to self-manage”* [34, 35] is more in line with older adults’ own perceptions of their health, as found in this study, than the former WHO definition that focuses on total well-being. The ability to adapt also relates to other orientations in gerontological successful aging theories such as “effective aging,” where older adults are not necessarily free from chronic diseases, but are able to adapt to the challenges that come with these diseases [36, 37].

Furthermore, older adults indicated that they experienced the interaction with the nurse as friendly and professional. In the interaction between the nurse and the older adult, certain skills and competencies such as listening, honesty, being supportive, and creating a positive atmosphere were highly appreciated. Related to these findings, clients of all age groups appreciate the following aspects of interacting with health professionals: 1) involvement in self-care, 2) independence, 3) being listened to, 4) mutual trust, and 5) sharing information [21, 31, 38]. More specifically, having someone showing an interest in one’s life who is able to listen to one’s story makes older adults feel valuable while they are aging, since someone cares about their perceived resources for improving their personal health [39].

Also, older adults felt they had been given professional advice which the nurse considered best practice during the consultations. There was significant variety in the way advice was given, which holds true for the decision-making processes as well. Only a few older adults said they talked about their personal considerations and preferences during the decision-making process. Only a few developed a shared goal or action plan to improve health-related behaviors during the consultations. It is, therefore, important to note that when referring to SDM during the consultations, it was only applied to a limited extent, which is in line with other studies examining SDM [40–42].

The third theme, in which older adults perceive the consultations as a physical check-up and /or a source of personal support, links to the other themes. The consultations in which older adults felt that the nurse was an expert who provided general advice on health-related topics, or where older adults experienced the consultation as a physical check-up, fit in with a more traditional public health approach. This approach includes disease prevention and a somewhat authoritative approach, in which the focus is on diagnosis and physical health, providing information to patients, and aiming for—sometimes even imposing—a change in patients’ behavior [6, 7]. Other older adults felt that they had received tailored advice, felt a connection with the nurse, and perceived the consultations as personal support. The latter experiences conform to a health promotion approach. In this approach, nurses take the individual’s perspective into account and, while working more collaboratively with patients, try to empower patients to make a change that favors their health status [6].

### Methodological considerations

The diversity of the interviewed older adults in terms of age, sex, and education, as well as their variety in views and perceptions, are the strength of this study. However, mainly native Dutch older adults were interviewed. The role of culture in relation to the nurse–client interaction could therefore not be included in the analyses. Further research is desirable to find out any cultural differences concerning this topic.

To ensure rigor, the authors have reflected on credibility, dependability, confirmability, and transferability [43]. To strive for credibility, the researchers wrote observations and narrative reports related to each interview consider the participant’s context. Dependability was achieved by using the approach of the step-by-step QUAGOL. Using the guide creates back-and-forth discussion with other internal and external researchers, which increases dependability. In addition, we formed a sound basis for further analysis by analyzing the first interviews independently with another researcher and discussing the outcomes [30]. To increase confirmability, an external senior qualitative researcher was asked to critically review the results and the ensuing critique was taken most seriously and implemented in the phase of describing the results for this article. Transferability was enhanced by using a purposive sampling method and providing a detailed description of our data collection method. In addition, the researchers used the consolidated criteria for reporting qualitative research (COREQ) to report important aspects of the research team, analysis, and interpretations of the data (S3 Table) [44].

### Recommendations

This study provides recommendations for practice, including nursing education. The individual experiences and holistic perspectives about healthy living seem to influence to what extent older adults are willing and able to change health-related behavior when needed. Thus, individual perceptions of healthy aging are an important starting point for the design of health promotion programs for older adults [45]. Additionally, it is important to empathize with older adults and find out what is important to them in healthy aging. It is a challenge for nurses to match their interventions to the current living situations of the older adults or to emphasize the importance of maintaining a healthy and independent life. Matching their expectations is important in this respect.

As part of nurses’ health promotion competencies [6], they should be better trained in communication skills (listening skills, creating an equal interaction, and SDM with the client). Aspects of MI such as evoking (eliciting older adult’s own motivations for change) and planning (formulating a concrete plan of action) deserve more attention in order to improve interaction between nurses and older adults in health promotion [14]. Also, decision talk, which refers to the task of arriving at decisions that reflect the informed preferences of older adults, guided by the experiences and expertise of the nurse, deserve more attention in nursing practice in order to avoid a missed match [46, 47].

## Conclusions

This study explored the views and experiences of older adults on their participation in a nurse-led health promotion intervention. It underscores the importance of matching nurse-led interventions to older adults’ personal views concerning healthy living, and to their views and experiences concerning these interventions. The study highlights their holistic view of health and their ability to adapt to the challenges that they face while growing older. The holistic views of older adults were not always assessed and valued by the nurses. In addition, this study shows a wide variety of expectations, views and experiences among the participating older adults. Ongoing attention is needed to teach students and professionals in communication skills in order to keep up with current person-centered care models.

## Supporting information

S1 Table Original quotations in Dutch with English translation. (DOCX)

S2 Table Minimal anonymized dataset. (DOCX)

S3 Table Consolidated criteria for reporting qualitative studies (COREQ): 32-item checklist. (DOCX)
