# Supplementary material for: Experiences and views of older people on their participation in a nurse-led health promotion intervention: “Community Health Consultation Offices for Seniors”
Source: PLoS One. 2019 May 13;14(5):e0216494. doi: 10.1371/journal.pone.0216494 (PMC6513064; doi:10.1371/journal.pone.0216494)
Supplement: S1 Table — (DOCX) [file pone.0216494.s001.docx]

**S1 Table 1.Original quotations in Dutch with English translation.**

| Dutch | English |
| --- | --- |
| “Nou gewoon, nou bijvoorbeeld. Ik heb mezelf voorgenomen elke dag moe je d’ruit. Je blijft niet de hele dag in de stoel, denk d’r om. Niet achter de geraniums. Die hebben we gelukkig niet meer maar goed (lacht). Maar nee. Hup! Buiten! (…) Al was het maar om een pak melk. Even naar de, effe naar effe naar de super.” - Quote 1 | “Well, you know, I promised myself that I would go outside every day. I tell myself, don’t sit in your chair all day, don’t even think about it! Don’t just sit there twiddling your thumbs. That’s no way to get a thumbs up (laughs). But anyway, chop chop, off we go! Outdoors! Even if it’s only for a carton of milk. Just a quick trip to the supermarket.”Quote 1 |
| “Gezond eet, nee gewoon gezond eten. Gewoon zo elke dag eh, dan zeggen ze weleens van. Dan moet je weleens invullen wat je allemaal ook eet. Dan denk ik het zal wel heel saai wezen. Het is elke dag hetzelfde, alleen de groentes wisselen elke dag he. Het is een dag in de week is het een keer macaroni en ik zeg altijd op vrijdag is het altijd bij ons de kliekendag, dan gaat de koelkast leeg.”- Quote 2 | “Eat healthily, no, just healthy food. Just like, every day. Then sometimes they ask you to fill in what you’ve been eating. Then I think it probably sounds very boring. It’s the same thing every day; it’s just the vegetables that change every day, you know. It’s once a week, it’s macaroni, and I always say Friday is leftover day, that’s when we empty out the fridge.”Quote 2 |
|  |  |
| Dutch | English |
| “En dat (spitten) is mijn hobby. En die geef ik nooit op. Want ik heb pijn hoor. Nou dan moet je even spitten, nou dan ga je even zitten. Hoppakee doorgaan. Doorzetten die handel (…) Dan ben je dus eh optimistisch maar ook koppig.” – Quote 3 | “Puttering around in the garden is my favorite hobby. I will never give that up. Even though I’m in pain, you know? I just dig for a while, and then I sit down for a while. Hop, and again. Just carry on. So, in that way you are optimistic, but stubborn too.”Quote 3 |
| “Dat je jezelf op je gemak voelt daar en dat je misschien eh..ook dingen zegt dat je misschien niet wilt zeggen bij wijze van spreken. Hebben wij niet gedaan hoor, maar dat zou je, dat als ik wat zou hebben wat ik denk ik aan een ander niet zou vertellen.” – Quote 4 | “That you feel at ease and maybe also eh...talk about things you wouldn't normally talk about, so to speak. Not to say we did that, but you felt you could. If there was something that I don’t think I would tell anyone else.”Quote 4 |
| “Ik zal nooit tegen een dokter of een ding zeggen dat ik zeg maar stoelgangkruiden gebruik, dat zal ik nooit zeggen want ik weet dat dat ook slecht is. Dat weet ik gewoon, alleen, ja je moet ook naar naar de/ naar achter gaan en als je dat niet kan zonder die dingen dan voel je je ook eh...ook rot. Nou en dat heb ik daar dus wel verteld en op een of andere manier geeft dat toch iets van eh toch vertrouwen of iets dat je daar dus dat wél zegt.(...) Stel dat ik met haar dus niet gezegd heb(...) dan zat ik er nou nog mee (...) Want ik wilde er vanaf maar ik wist niet hoe. Ik denk ik kom er nooit vanaf.” – Quote 5 | “I would never tell any doctor or medical person that I use herbal remedies .I would never tell them because I also know it’s bad. I just know. But yes, you also have to go to the bathroom and if you can’t without those things then, then, you also feel eh, rotten. Well and I did talk about it then (to the nurse during the consultation) and somehow that does give you some confidence or something, to talk about it to the doctor. If I hadn’t told the nurse, then it would still be a problem now. Because I wanted to stop but didn’t know how. I thought I would never be able to stop (using these herbal remedies ).”Quote 5 |
| Dutch | English |
| “Eh laat ik het zo zeggen, de adviezen die ik kreeg werden dus sterk aangeraden omdat ze zei nou dat is eh..de beste manier om te doen. Nou, ze liet ze doorschemeren, als je het zo doet is het verstandig, als je het niet doet is het onverstandig. Maar goed de keuze is aan jezelf, ze kan je niet dwingen.” Quote 6 | “Let me put it like this, the advice I got was strongly recommended because the nurse said, well, that’s the best way to do it. Well, the nurse gave the impression; if you do it like that it’s wise, and if you don’t it’s foolish. Anyway, the nurse said to me, the choice is yours. The nurse can’t force you.” Quote 6 |
| “Zij doet haar werk. En zij wil ook het goeie voorbeeld geven dat het dus beter voor je is om het niet te doen. Heb ik alle begrip voor. Maar, daar had ik dus geen interesse in. (lacht)” – Quote 7 | “She’s doing her job. And she also wants to set the right example, that it’s better for you not to do it (smoking). I get it. But it didn’t interest me at all (laughs).” Quote 7 |
| “Ja, ja 't is, misschien vind ik het dan wel niet zo prettig meer. Dat dat je zegt ik ga er niet meer naartoe! Want ik heb/ dan komt ze weer met dat gezeur aan met je bent wel wat aan de zware kant of eh, en je moet dit en je moet dat, dat zeggen ze dan. Je moet dit en je moet dat en je moet zus en je moet zo, weet je wel, dan zitten ze je ook wel een beetje de les te lezen he.”- Quote 8 | “Because the nurse starts nagging again saying I might be a bit on the heavy side or uh, you have to do this or that. That’s what they say. You have to do this and you have to do that, you know, then they are really lecturing you a bit, you know.” Quote 8 |
|  |  |
| Dutch | English |
| “Want je wil niet in je hemd staan. Niemand/nee want als je wat belooft en je en je komt het niet na, dat vind je niet leuk of wel dan. En ik bedoel maar/ en als je dan eh, ergens heen gaat, dan heb je natuurlijk wel, een belofte maken/ dan heb je wel een stok achter de deur om te zeggen nou ik heb het haar beloofd. Dan moet ik (…) Want we hadden wel heel goed contact samen. Ik denk nou dat dat krijg ik voor mekaar weet je wel. Ik denk dat doe ik weet je wel (...). Want dat was ook mijn streven, want omdat eh/ ja ze kwam heel vriendelijk over. Ik denk nou dat eh, dat redden we dan wel.” – Quote 9 | “Because you don’t want to look like a fool. If you make a promise and you don’t follow through, that just doesn’t feel right, does it? And I mean, if you go somewhere, then you have to make a promise. Then you have the motivation to say, ‘well, I did promise the nurse. Well, then I just have to.’ I’m thinking, ‘well, I can manage this, you know.’ I’m thinking, ‘I’m going to do this,’ you know. Because that was also my goal. I’m thinking, ‘well that eh, we can make this happen.” Quote 9 |
| “Genoeg, die APK is meer als genoeg.” – Quote 10 | “Yes, sufficient. That check-up is more than sufficient.” Qoute 10 |
| “Nee...toegevoegde waarde...Nou ik vind als het gezellig is, nou dan is het toch goed?” – Quote 11 | “ Added value..Not really... Well, I think if it’s a nice chat, then that’s okay right?” Quote 11 |
|  |  |
| Dutch | English |
| “Ja, we hebben wel stilgestaan tuurlijk bij mijn man. He, dat is echt aandacht. Omdat ik daaronder leed. Maar dat heb ik nooit in de gaten gehad dat ik d’r eigenlijk zo onder leed. He dat het een hele grote plek in nam in mijn leven he? (...) Maar weetje, je moet wel, van buitenaf, moeten ze juist, de juiste eh.. ja.. eh..draadjes zien te vinden bij je he. Van weet je het moet net aangetikt worden, want voor iemand anders geldt heel wat anders dan voor mij. Terwijl ik zelf heel goed mijn mondje kan roeren, maar je hebt op een gegeven moment als je met een man leeft die bijvoorbeeld depressief is, dan ga je heel geleidelijk aan in dat proces mee. He. En dat is doorbroken gewoon, dat ik dat niet hoefde te doen. Dat ik het anders moest aanpakken.” – Quote 12 | “Yes, we did talk about my husband. That’s real attention, you know. Because, I was suffering because of that. But I never noticed that I was suffering actually. That it was such a big part of my life you know? But you know, you do have to from outside, they have to find your right, well, the right triggers, you see? It almost happens by chance, because not everyone is the same as I am. Despite me being pretty chatty myself, but at a certain point when you’ve been living with a depressed husband, you get pulled into that process gradually, you know. And that has just been a breakthrough, that I didn’t have to do that. That I should find another way to deal with that.” (Female, age 70) – Quote 12 |
