# Supplementary material for: Experiences and views of older people on their participation in a nurse-led health promotion intervention: “Community Health Consultation Offices for Seniors”
Source: PLoS One. 2019 May 13;14(5):e0216494. doi: 10.1371/journal.pone.0216494 (PMC6513064; doi:10.1371/journal.pone.0216494)
Supplement: S2 Table — (DOCX) [file pone.0216494.s002.docx]

**S2 Table 2. Minimal anonymized dataset.**

| **Themes and categories** | **Codes** | **Example quote in Dutch** |  |
| --- | --- | --- | --- |
| **Theme 1: Awareness of aging** |  |  |  |
| *A healthy lifestyle* | Food and drink | Gezond eten, nee gewoon, gezond eten. Gewoon zo elke dag... eh, dan zeggen ze weleens, dan moet je weleens invullen wat je allemaal eet. Dan denk ik het zal wel heel saai zijn. Het is elke dag hetzelfde, alleen de groentes wisselen elke dag, hé? Het is één dag in de week een keer macaroni en ik zeg altijd, op vrijdag is het altijd, bij ons, de kliekendag. Dan gaat de koelkast leeg. |  |
|  | Mental wellbeing | Nou, ik geloof nou toch wel dat is iets... dan moet je niet dement worden of eh...nou ja. (…) Dat is, dat vind ik wel gezond ouder worden. Als je het koppie erbij houdt. |  |
|  | Mindfulness | Tai chi doe ik aan mee. En ehm, van die mindfulness heb je ook heel veel huisoefeningen en ik zal u eens zeggen dat ik op het ogenblik... Ik deed het heel trouw en ik wil dat eigenlijk ook [blijven] doen, omdat ik zelf denk, bij het ouder worden is dat belangrijk. |  |
|  | Alcohol in moderation/no alcohol | Maar dat eh, dat doe ik ook niet. Ik heb altijd twee glazen wijn per dag gedronken en nu drink ik nog een half glas wijn 's middags om vijf uur. |  |
|  | Conscious of unhealthy lifestyle | En, maar ik ben ik ben te dik hoor, ik ben te dik. Ik mocht wel 10 kilo lichter zijn maar ja, daar moet ik een klein beetje beter aan werken. |  |
|  | Physical activity | Nou gewoon, nou bijvoorbeeld. Ik heb mezelf voorgenomen, elke dag moet je eruit. Je blijft niet de hele dag in de stoel. Denk erom. Niet achter de geraniums. Die hebben we gelukkig niet meer maar goed (lacht). Maar nee. Hup! Buiten! Als het (onverstaanbaar), dan ga jij naar buiten. Al was het maar om een pak melk. Even naar de...even naar, even naar de super. |  |
|  | Rest, cleanliness, and regularity | Je bent het zo gewend van vroeger eigenlijk al. Thuis was het ook altijd eh, regelmaat. En eh, en alles op orde. Dus ik ben niet dat je zegt nou, ik zit er, erover uit en ik ga dit doen of ik ga dat doen, dat heb ik niet. |  |
|  | Sleeping | Maar toch op tijd naar bed gaan. Het is eh, om tien uur een beetje klarigheid maken en om elf uur lig je in bed. |  |
|  | Not smoking | Het is dus een kwestie van niet meer roken. Daar heb ik... ik heb het wel gedaan. Heb ik ook opgegeven. En ook met nog meer, ten behoeve van, ook de kleinkinderen en noem maar op, van Jan en alleman. |  |
|  | Social contacts | Nee, ik ga ook wel de stad in en...Nou ja, en die sociale contacten zijn nog wel goed hoor. Dus niet dat je de hele dag binnen zit. |  |
|  | Mobility | Dat ik mezelf eh, een beetje goed voort kan bewegen nog en eh... Maar dat lukt op het moment niet, want ik ben een beetje onder doktersbehandelingen. |  |
|  | Being an example to others | Ik vind gewoon, als je ouder wordt he, dan eh, dan moet je gewoon zorgen dat je een voorbeeldfunctie hebt, naar de hele maatschappij toe. Ook naar je kinderen, naar je kleinkinderen. Van eh, hoe je gedrag hoort te zijn. Hè, kijk, wij zitten gewoon, wij hebben het gehad! Wij hebben onze, wij hoeven alleen nog maar leuke dingen te doen. Ik vind, en daar moet je als oudere aan denken. Dat je gewoon lief bent voor elkaar, dat je elkaar waardeert en respecteert. |  |
|  | Enjoyment | Dat ik van het leven mag genieten eigenlijk! [lacht] |  |
|  | Hobby’s | Nou dat zeg ik, biljarten. |  |
| *Dealing with (new) opportunities and constraints in life* | Activate yourself | Hè, het kan niet allemaal tegelijk natuurlijk. Dus eh, ja.. dan ehm, heb ik weleens eventjes de neiging om te denken van eh.. (zucht). Hè, gaat het nou wel goed, en eh...enzovoort hè? Dus dan ben ik eventjes een beetje...Maar niet dat ik daar verder in mijn denken ehm..dat ik zo denk weet je wel, zo. [...] Ik zit mezelf ook weleens eventjes op te voeden [lacht]. Nou gewoon, dan zeggen van eh, niet pessimistisch zijn. |  |
|  | Perseverance | Als je tien meter loopt, moet je tien meter terug. En dat heb ik heel goed in mijn hoofd geknoopt. Dus wat ik vandaag niet kan, kan ik morgen wel. En wat ik morgen kan, kan ik overmorgen weer een stukje verder. |  |
|  | Aspiring to a long life | Ja, ik eh, nou ja, bij wijze van spreken wil ik 100 worden. |  |
|  | Being positive/optimistic | Proberen heel positief in het leven te staan. |  |
|  | Anticipation, Acceptance | Kijk, ik vind [dat] als je ouder wordt, moet je gewoon alles he, eh, gaan minderen. Je moet hè, je moet je niet vol eten [en] op een stoel gaan zitten. Dat wordt gewoon helemaal niks. Nee. |  |
|  |  | Gezond ouder worden. Dat ik accepteer dat eh, dat dat er dingen minder kunnen. En, maar dat er vaak wel andere mogelijkheden ontstaan |  |
|  | Knowledge/life experience | Nou, en wij doen het zo en dat bevalt uitstekend. [...] Maar dat komt ook omdat je volgens mij eh, gezonde mensen bent. |  |
|  | Nothing is a must, anything goes | s Morgens als je uit bed komt, er is geen moeten, het mag allemaal. |  |
|  | Unpredictability of life | Dat eh, dat weet je toch niet, wat er in je lijf gebeurt of [wat er] in je hoofd gebeurt. Dus dat weet je niet. |  |
|  | Seize the day | Als ik 's morgens wakker word, dan ben ik heel blij al, dat ik de dag weer aanschouwen kan. |  |
|  | Take your responsibility | Maar er hoort wel bij dat je zelf aan je gezondheid moet werken. Kijk, wanneer je dood gaat, dat is bepaald. Maar de kwaliteit van leven, dat bepaal je zelf denk ik. |  |
|  | Continuing doing the things you always did | Nou ja, gewoon dat ik, dat, ik red het nog steeds! […] Dus, voor de rest, ja sta ik er eigenlijk niet bij stil. |  |
|  | Religious coping activities (e.g. prayer) | Maar eh, ik hoop eh, dat door gebed [...] dat ik geen gehoorapparaatje [hoef]. Eh, dat de Heer een hoop geld voor me uitspaart. |  |
| **Theme 2: Experienced interaction with the nurse** | |  | |
| *Evaluation of the nurse’s general skills* | Asking questions versus not asking questions | Zoiets van, ik heb iemand om mij heen gehad die helemaal... En die ook dan ook vragen stelt weet je wel. Waar je dan op zo'n moment antwoord op kunt geven. Dus ja, dat eh, dat vind ik eh, dat vind ik gewoon fijn. |  |
|  | Advocacy | Als er iets is dat het bespreekbaar is en dat ze.... Ik had het gevoel dat ze voor je opkomen. |  |
|  | Empathy | Nou ik denk dat ze, een eh, hoe heet dat, een invoelings-, eh...eigenschap hebben. |  |
|  | Supportiveness | Vertellen dat je dat gewoon heel rot vond, bepaalde dingen. Hè, dat je het rot vond. Ik heb daar ook wel zitten huilen, een beetje. Dat ik soms zei, [ik] ben machteloos. ‘Hè, het hoeft echt niet. We gaan u daarbij helpen. En ik laat je niet in de steek’. |  |
|  | Listening, to be taken seriously versus not feeling taken seriously | Aan haar opmerkingen kon je merken dat ze ook echt luisterde. Dus het was niet zo eh, nou ja het gaat me er hier in en er daar weer uit. |  |
|  | Personal approach versus impersonal approach | Je had niet het gevoel dat je als een nummer behandeld werd. Je werd als een persoon behandeld. |  |
|  |  | Nee gewoon, dat ik er toevallig was, dingen werden opgeschreven. |  |
|  | Creating a positive atmosphere (friendly, open, making people feel at ease) | Maar wat mij het meest is bijgebleven. dat is gewoon, nou, de vriendelijkheid. |  |
|  | Honesty | En ze is wel eerlijk geweest met ja, dat ligt niet op mijn pad. |  |
|  |  | Als ik een vijandig gesprek inga, weet ik niet hoe zij dan overkomt. Maar ja goed, als ik gewoon daar naartoe ga, open en eerlijk, dan krijg ik van haar ook een open en eerlijk antwoord terug. |  |
|  | Reflection/evaluation | Dat heb ik toen ook gezegd; oh ja, ik denk het haast [wel]. Ik denk dat ik ook, toen dat hij even vroeg wat vond u ervan, dit gesprek...En toen zei ik nou heel prettig en fijn dat eh dat eh,... prima |  |
|  | Competent nurse versus incompetent nurse | Laat ik het zo zeggen, want eh als je iemand neerzet die alleen maar de vragenlijst beantwoordt, daar schiet je niks mee op. Dus zulke mensen met praktijkervaring die weten waar ze het over hebben. En die mensen moet je daar laten [neerzetten], die kunnen je dan ook advies geven op maat. En niet eh.. alleen eh via het boekje. |  |
|  |  | Het was net alsof zij haar taak niet helemaal goed verstond... |  |
|  | Connection versus no connection | Van die andere [verpleegkundige] denk ik nou ja, ik vind het wel goed, maar voor haar, ik denk nou... Want we hadden wel heel goed contact samen. Ik denk, nou dat krijg ik voor elkaar weet je wel. Ik denk dat doe ik, weet je wel? |  |
|  |  | Nah, nee dat eh, voor de rest heb ik er niks mee […] Zelfde net als dat je 's morgens eh gaat bloedprikken” |  |
| *Receiving advice* | Receiving information during the consultation | Want eh, je wacht soms te lang. Om naar de dokter te gaan. Dan denk je oh, dat valt misschien nog wel mee. En als er dan zo’n consultatiebureau is en die geeft je wat richtlijnen, voorlichting, en die zegt van ’t is beter zus of zo of dit of dat, dat is, dan kun je altijd nog naar de dokter gaan. |  |
|  | Content of advice | Nou ja, dat ik dus, dat [het] heel goed zou zijn als ik wat zo afvallen. Dat was dus advies één. |  |
|  |  | En wat, wat zij voorschreef eh, bewegen en afvallen... |  |
|  |  | Dat kan ik mij zo niet herinneren, dat ik nou zulke adviezen heb gehad. |  |
|  |  | De heer rookt eh...Advies met het roken. Hulp proberen, eh proberen te krijgen met stoppen. |  |
|  |  | Zo, u moet eh, activiteiten hebben, niet de hele dat thuis zitten en wat doen. En dat doe ik. |  |
|  | Referrals | En eh, zij heeft mij dan toen doorgestuurd naar een diëtiste. |  |
|  |  | Ja, hij zei toen van, ‘vindt u het goed dat (voornaam) (achternaam) komt, voorzitster van het Alzheimer café?’ En zij is dus verpleegkundige op het gebied van dementie. ‘Vindt u het goed dat zij bij u’, vroeg ie, ‘of zij bij u thuiskomt’? |  |
|  | Written advice received from the nurse | Dat is voor mij. Dat advies wat ze mij dus eh... Kijk dat krijg je dan mee... [geeft advies kaartje aan ons] |  |
|  |  | Heb ik niet gekregen |  |
|  |  | Is wel een hele poos geleden hè. Zal ik wel weggegooid hebben. Ik heb hem niet meer. Want het is natuurlijk al weer heel wat maanden geleden […] En dan ruim ik die rommel dan weer even op. |  |
|  | Way of advising | Benoemen = observatie maken: Eh, nee zij concludeerde alleen eh dat die BMI, en bij oudere mensen eh, anders is. |  |
|  |  | Bevragend = vragen of optie geschikt zou kunnen zijn: Nou en eh toen zij ze van, ‘ja, nou misschien dat u in plaats van twee glaasjes wijn één glas wijn per dag kunt drinken’ |  |
|  |  | Info/voorlichting = algemene (niet op maat) voorlichting gegeven: Ik heb alleen de schijf van vijf meegekregen, de allereerste keer. Ik kan hem zo pakken dus eh, [lacht], ja! |  |
|  |  | Dwingend = autoritair als in: je moet dit of je moet dat: ‘Ja, maar u moet meer vis eten!’ U hebt mooi pra[ten]. Ik hou niet van vis! Alleen de lucht al! |  |
|  |  | Verstandige keuze/aanraden = verpleegkundige raadt sterk aan om iets te doen, legt maar 1 ‘best practice’ keuze voor: Het is niet zo dat eh ze, de [verpleegkundige], dat ze het dwingend zei maar, ze raadde het wel sterk aan. Laat ik het zo zeggen. |  |
|  | Follow-up | Ik heb er eigenlijk niks mee gedaan. Ja ik heb hem [advieskaart] even doorgekeken. Toen dacht ik ja maar meid, ik... Wat moet ik hieraan veranderen! Ik eet elke morgen hetzelfde. Ik heb opgegeven wat ik at. |  |
|  |  | Ja ik ben eigenlijk al veranderd, ik ben naar de diëtiste gegaan, ik doe toch bepaalde dingen die zij wel zegt. |  |
| *Decision-making processes* | Participation and power balance | Ja, ik vond het gelijkwaardig. |  |
|  | Respecting autonomy | Ze liet duidelijk merken ‘het is uw eigen keuze, maar als u het niet doet is dat niet zo verstandig. Verstandiger is om het wél te doen’. Dus eh, het is op een vrijblijvende manier, maar op een hele nette manier. |  |
|  | Presentation of options and priorities by the nurse | Ja, nou laat ik het zo zeggen, ze gaf gewoon de mogelijkheden die er waren, en dan moet je zelf een keuze maken ,wat je kan. |  |
|  | Exploring personal preferences and options | Toen dacht ik, ja ik vind het gewoon heel erg lekker! Toen dacht ik, maar ja, het is ook zo... je hebt het niet nodig. Dus ik kan dat gewoon doen. Nou, en nu neem ik dus een half glaasje om vijf uur. En als ik zin heb drink ik nog een half glaasje. |  |
|  | Discussing the problem | Geen motivatie bespreken ongezond gedrag = client wil probleem niet bespreken met verpleegkundige: Daar kunnen we het beste... Dan zegt ze... Nou kunnen we dan...Ik zeg, kun je ook, hoef je het ook niet over te hebben want ik stop er [roken] niet mee |  |
|  |  | Probleem besproken (tijdens consult) = probleem is besproken (en meer niet): Nee, ik heb echt tegen haar gezegd, ik ga er [overgewicht] aan werken maar ik weet niet op dit moment wat ik moet doen om dat [extra gewicht] eraf te krijgen. |  |
|  | Precontemplation (not open to changing behavior) | Als je er verder eh, aardig goed bij voelt en je bent wat aan de zware kant, eh, waarom zou ik me er dan verder druk om maken? |  |
|  | Former failures to change behavior | Ja dat probeerde ik ook [gedrag veranderen]. Ja dat probeer je altijd wel, maar dat gaat, dat is niet zo eenvoudig hè? |  |
|  | Keeping true feelings/thoughts to oneself | Verpleegkundige gelijk geven/meepraten: O ja ja, want dat heb ik toen niet verteld. Want ik gaf er ook niet weer om. |  |
|  | Making choices together | Want je wil niet in je hemd staan. Niemand/nee want als je wat belooft en je en je komt het niet na, dat vind je niet leuk of wel dan. En ik bedoel maar/ en als je dan eh, ergens heen gaat, dan heb je natuurlijk wel, een belofte maken/ dan heb je wel een stok achter de deur om te zeggen nou ik heb het haar beloofd. Want we hadden wel heel goed contact samen. Ik denk nou dat dat krijg ik voor mekaar weet je wel. Ik denk dat doe ik weet je wel. Want dat was ook mijn streven, want omdat eh/ ja ze kwam heel vriendelijk over. Ik denk nou dat eh, dat redden we dan wel. |  |
| **Theme 3: Perception of the consultations as check-up and/or personal support** | |  | |
| *Perceiving the consultation as a physical check-up* | Focus on physical health | Ja, zo zie ik het. Als een bepaalde lichamelijke keuring. |  |
|  | Control function | Je wordt een beetje in de gaten gehouden. Zeg maar, van het gewicht, zus zo, dat dat, al die dingen. |  |
|  | Feeling of security | Dat als ik wat zou hebben wat ik denk ik aan een ander niet zou vertellen. |  |
|  | Second opinion | Ja. Nou ja goed, ik benoem het...Dan kan je een second opinion ten opzichte van iemand anders [krijgen].. |  |
|  | No influence on personal lifestyle | Ik drink nog net zoveel koffie. En ik snoep niet meer en ik snoep niet minder [lacht]. |  |
|  |  | Gaat hier erin en daar eruit (lacht). (...) Zoveel indruk heeft het niet gemaakt. En eh, voor mij heb ik het idee dat ik toch een eh, goed le/ ja goed leven heb. Gezond leven leid en dergelijken en dat ik mijn best doe om zo verder te gaan. |  |
|  | Positive impact on lifestyle | Nu zit ik wel aan het gewicht wat ik haar beloofd had. Dus ik heb nu wel eh, nu ben ik wel een beetje afgevallen. |  |
|  | Raising awareness | Je wordt wel met de neus op de feiten gedrukt ondanks dat je daar geen belang achter zet. Ze zegt ook ‘het kan nu nog wel goed gaan, maar dat blijft hoogstwaarschijnlijk niet goed gaan’. Snap je wat ik bedoel? |  |
|  | Confirmation of healthy behavior/health status | Nou gewoon dat je weet, hè, je bloeddruk is goed, want die veel te hoog is geweest. Dat weet ik nu zonder dat ik naar mijn huisarts toe hoef. Dit is een vrijblijvender iets. |  |
|  | Informative | Ik kom nu bijna niet meer bij mijn huisarts, omdat ik goed voorgelicht wordt bij het consultatiebureau voor ouderen. |  |
| *Receiving personal support from the nurse* | Focus on mental health | De verpleegster is ook opgeleid om eventueel geestelijke vragen te beantwoorden, of samen een oplossing te gaan zoeken. Kijk dan wordt het wat anders. |  |
|  | Good feeling | Ik ging ook echt eh..met een eh..heel fijn gevoel ging ik eigenlijk weg. |  |
|  | Breaking off fixed patterns | In een levensritme, waarin je heel lang in zit. En dat je leert dat te doorbreken. Ik vind het gewoon heel jammer dat mijn man het niet doet. Dat je gewoon leert, iets hè, te doorbreken. Of los te laten. Voor jezelf op te komen. Of dat er heel veel dingen zijn veranderd en dat je daar best over mag praten. |  |
|  | Empowerment | En dat ik daar nóg weer meer, voor gemotiveerd ben. En dat wil ik ook echt het laatste...Ik ben aan de laatste fase van mijn leven bezig. En ik vind het heel fijn dat ik ehm, daar in mijn laatste levensfase mee bezig kan. |  |
|  | Feeling of support | Maar wat het belangrijkste eigenlijk was van die sessies, dat vond ik zelf, dat eh, dat ik een gevoel had dat ik ergens in gesteund werd. En dat vond ik heel prettig. |  |
|  | Someone who is listening/being able to tell your story | En ik kan ook mijn verhaal kwijt. Kijk dat, ik geloof dat dát het allerbelangrijkste is. Dat wij ons verhaal kwijt kunnen. |  |
|  |  | In de totaliteit, alleen het gevoel: er wordt naar je geluisterd. |  |
|  | Moment of personal contact | Nee...toegevoegde waarde...Nou ik vind als het gezellig is, nou dan is het toch goed?” |  |
|  | Having someone to fall back on | Kijk en dan denk je van nou ja gut, er kunnen wel dingen voorkomen, voorvallen. Je weet het niet, dan denk je, nou kun je bij de verpleegkundige ook, vraag ik haar es naar. |  |
